# Recent Technology-Aided Programs to Support Adaptive Responses, Functional Activities, and Leisure and Communication in People With Significant Disabilities

**DOI:** 10.3389/fneur.2019.00643

**Published:** 2019-07-02

**Authors:** Giulio E. Lancioni, Marta Olivetti Belardinelli, Nirbhay N. Singh, Mark F. O'Reilly, Jeff Sigafoos, Gloria Alberti

**Affiliations:** ^1^Department of Neuroscience and Sense Organs, University of Bari, Bari, Italy; ^2^Interuniversity Center for Research on Cognitive Processing in Natural and Artificial Systems (ECONA), Sapienza University of Rome, Rome, Italy; ^3^Medical College of Georgia, Augusta University, Augusta, GA, United States; ^4^Department of Special Education, University of Texas at Austin, Austin, TX, United States; ^5^School of Education, Victoria University of Wellington, Wellington, New Zealand; ^6^Lega F. Doro Research Center, Osimo, Italy

**Keywords:** technology-aided programs, support tools, disabilities, cognition, adaptive responses, functional tasks, leisure, communication

## Abstract

This paper presents an overview of recent technology-aided programs (i. e., technology-aided support tools) designed to help people with significant disabilities (a) engage in adaptive responses, functional activities, and leisure and communication, and thus (b) interact with their physical and social environment and improve their performance/achievement. In order to illustrate the support tools, the paper provides an overview of recent studies aimed at developing and assessing those tools. The paper also examines the tools' accessibility and usability, and comments on possible ways of modifying and advancing them to improve their impact. The tools taken into consideration concern, among others, (a) microswitches linked to computer systems, and aimed at promoting (i.e., through positive stimulation) minimal responses or functional body movements in individuals with intellectual disabilities and motor impairments; (b) computer systems, tablets, or smartphones aimed at supporting functional activity engagement of individuals with intellectual disabilities or Alzheimer's disease; and (c) microswitches with computer-aided systems, elaborate communication devices, and specifically arranged smartphones or tablets, directed at promoting leisure, communication, or both.

## Introduction

Technology-aided programs are increasingly recognized as essential means for supporting people with significant disabilities (e.g., congenital intellectual, motor, or sensory impairments, and possible combinations of them, and neurodegenerative or post-traumatic disorders) within their daily contexts (1–5). Technology-aided programs are designed to build functional links between user, technology and environment, and thus ensure that a satisfactory (goal-directed) interaction with the environment is possible also for individuals with extensive levels of disabilities (6). Such interaction is considered critical to promote the individual's general achievement, personal satisfaction (quality of life), social image, and cognition (2, 7–10).

Technology-aided programs for people with significant disabilities have largely focused on providing them with support in main problem areas, such as passivity and detachment, failure to carry out functional activities, and failure to engage in leisure and communication. The first area (i.e., passivity and detachment) is concerned with the inability to engage in simple responses/movements functional to interact with the environment and, possibly, carry out forms of physical activity with potential health benefits (4, 11–14). For example, people with pervasive motor impairments and intellectual disability (or consciousness disorders) tend to be passive and detached and thus fail to reach any control of environmental stimulation and improve their general alertness and awareness (12, 15). Similarly passive and detached may also be people with severe/profound intellectual disabilities who have less extensive (or no specific) motor impairments, as well as people who are affected by advanced Alzheimer's disease (4, 16, 17).

The second area (i.e., failure to carry out functional activities) is concerned with the inability to engage in complex, relevant tasks, such as vocational, domestic, and self-care tasks. For example, people with moderate intellectual disabilities frequently fail to independently perform vocational tasks because they cannot remember the steps and materials involved in those tasks (18, 19). The same people as well as people affected by mild/moderate Alzheimer's disease or acquired brain injury may be unable to independently perform relevant domestic tasks because they do not recall the time of the day when those tasks are due and/or the task steps (20–22).

The third area (i.e., failure to engage in leisure and communication) is concerned with the inability to manage leisure activities and communication interactions independently. For example, people with severe intellectual and developmental disabilities may be unable to start and engage in leisure activities on their own and thus remain dependent on staff or caregivers (23–25). The same people may also be unable to communicate their needs or desires, that is, to make clear requests to and have some basic interaction with communication partners (i.e., staff and family members) present in their immediate surrounding or distant from them (26–29). Serious leisure and communication problems also occur among people with neurodegenerative diseases (e.g., amyotrophic lateral sclerosis) and post-traumatic multiple disabilities (30–33).

Technology-aided programs set up to address the aforementioned problem areas are generally designed to function as support tools aimed at bridging the gap between the individual's actual skills and the skill level required to reach meaningful goals. Support tools can be expected to work effectively only if arranged in line with the individual's specific condition and the goal set for him or her. For example, if the individual's specific condition is severe intellectual disability and extensive motor impairment, support tools might be designed to help him or her carry out various forms of adaptive responses. In practice, support tools might help the individual to (a) make one or few small responses available in his or her repertoire effective to produce a relevant environmental change (i.e., a change that the response or responses *per se* would be inadequate to produce), or (b) use a functional body movement to reach relevant environmental events and, at the same time, manage some form of mild, potentially beneficial physical exercise (4, 12, 34).

If the individual's specific condition is moderate intellectual disability or mild to moderate Alzheimer's disease with inability to carry out basic/functional daily tasks, support tools might be designed to provide (a) time cues (i.e., reminders when the tasks are due), and step instructions (i.e., verbal or pictorial instructions concerning the material and responses required for each single step of the task to carry out) (20, 35, 36). Such tools would allow the individual to have a positive role in the environment and to gain social appreciation and respect (34).

If the individual's specific condition is moderate intellectual disability with sensory and/or motor impairments, or emergence from a minimally conscious state with motor and speech disabilities, support tools might be designed to promote leisure engagement, communication, or both. In the first case, the support tools would serve to present the individual with different leisure options and allow him or her to choose among them with simple responses (24, 37). In the second case, the support tools would serve to offer communication options (e.g., the opportunity to make verbal requests or send text messages) and allow the individual to choose among those options and activate them (29). In the third case, the support tools would serve to offer the individual leisure and communication options. The individual would then be allowed to choose between those types of options as well as among the alternatives available within each option (24, 38).

This paper is an effort to illustrate some of the support tools mentioned above (i.e., in relation to the three main problem areas) by providing an overview of recent studies aimed at developing and assessing those tools. Given the specific, and rather circumscribed, scope of the paper (i.e., as just indicated) the tools illustrated and the studies reported to describe their applicability and impact represent a non-systematically selected group of the tools and studies available. The selection was made by the authors based on two simple, intuitive criteria. The criterion used for selecting the tools was their perceived technical and practical relevance. Essentially, the tools selected were deemed to represent innovative (challenging) and effective resources for fostering favorable changes within the main problem areas identified above. The criterion used for selecting the studies concerned the fact that they represented recent evidence in the field (i.e., had been published within the last few years) and provided clear illustrations of the tools' technical aspects and support potential. In summary, the paper describes the characteristics and impact of a series of support tools (i.e., the technology components involved, the intervention strategies set up to make those technology components an integral part of the individuals' response/interaction with the environment, and the results obtained) with the help of studies reporting such tools. The paper is also providing comments on the tools' accessibility and usability, and possible ways of modifying and advancing them to improve their impact.

## Support Tools to Promote Adaptive Responses

As suggested above, adaptive responses may involve (a) small/minimal movements (e.g., small limb or head movements as well as finger, lip, and eyebrow movements) (14, 39) and (b) large and functional body movements (e.g., leg lifting and ambulation steps) (4, 17, 40). Minimal movements/responses are not considered suitable to allow the individual to have any impact on the environment or produce some form of physical exercise useful for his or her condition. Yet, the same responses may become critical to enable the individual to interact with the environment and control relevant events if adequate support tools are used within a suitable intervention process (13, 14, 41). Functional body movements can be instrumental to control environmental events and, at the same time, can constitute beneficial forms of physical exercise (4, 12, 17, 40). Making minimal responses or functional body movements instrumental to control environmental events is critical for two reasons. First, it may be the only way to enable the individual to reach those events and control their occurrence independently. Second, the occurrence of those events can motivate the individual to repeat the emission of the responses/movements over time and thus ensure consistent contact with the environment, arousal and engagement, and possibly relevant physical exercise (6, 40, 42–44).

The support tools required to help individuals develop and strengthen small responses or functional body movements and make them relevant consist of devices that can (a) monitor the emission of those responses and (b) ensure that they trigger the occurrence of environmental events preferred by the individuals (e.g., music, videos, and familiar voices addressing them). Six recent studies are summarized in this section of the paper to provide (a) illustrative examples of the support tools available for fostering small responses and functional body movements, and (b) an informative basis for commenting on those support tools (12, 16, 44–46). Two of the six studies were specifically directed at individuals who could only produce small responses (12, 45), while the other four studies were directed at individuals who could perform functional body movements (see Table 1).

**Table 1:** Studies using support tools to promote small responses and functional body movements.

**Responses/Authors**	**Participants**	**Age**	**Support tools**
	**number**		
**SMALL RESPONSES**			
Lancioni et al. (12)	4	7−34	Microswitch linked to a computer ensuring that each target response led to stimulation
Lancioni et al. (45)	10	25–81	Microswitch linked to a computer ensuring that each target response led to stimulation
**FUNCTIONAL BODY MOVEMENTS**			
Shih and Chiu (48)	2	16, 17	Dance pad linked to a computer and a television set ensuring that in place walking led to stimulation
Stasolla et al. (44)	2	5, 6	Microswitch linked to an electronic control system ensuring that each ambulation/step response led to stimulation
Lancioni et al. (16)	11	72–91	Microswitch linked to a computer ensuring that each leg response led to stimulation
Lancioni et al. (46)	7	9−42	Smartphone monitoring specific head, arm or leg responses and ensuring stimulation at the occurrence of such responses

### Small Response*s*

#### Studies

The first study dealing with small responses (12) focused on the development and assessment of a support tool relying on microswitches for four participants who were affected by a massive paralysis of their body and allegedly severe/profound intellectual disability, subsequent to congenital encephalopathy, or spinocerebellar ataxia. None of the participants had any form of communication or interaction with the environment. Two participants used lip movements as their response. The other two participants used prolonged eyelid closure and eyelid opening, respectively. The responses were monitored via an experimental optic microswitch involving an infrared light-emitting diode and a mini infrared light-detection unit fixed on the participant's face or via a camera-based microswitch placed in front of the participant. Each response activated the microswitch, which in turn triggered a computer that delivered 10 s of preferred stimulation (e.g., audio and video recordings with music and familiar voices). All participants showed a clear increase in response frequencies when the support tool was in use.

The second study (45) focused on the application and assessment of a microswitch-based support tool similar to that described above with 10 participants who were in a minimally conscious state and presented with extensive motor impairment and lack of speech or any other functional communication following brain injury and coma. Their responses included eyelid closures, and small head, hand/finger, foot, or lip movements. Eyelid closures and lip movements were detected via optic microswitches (such as the one mentioned for the previous study). Head and foot responses were detected through simple pressure microswitches. Hand or finger movements were detected through microswitches sensitive to touch and pressure. As in the study summarized above, each response activated the related microswitch and this triggered a computer, which delivered 10 s of preferred stimulation. All participants showed meaningful increases in their responding during the intervention phases of the study in which the support tool was in use, thus showing improved levels of alertness, attention, and activation.

#### Comments on the Support Tools

The support tools described above can be considered the most immediate instruments for enabling individuals with pervasive impairments to have self-determined contact with the environment, control their stimulation input, and improve their social image (2, 47). From a technical standpoint, it should be underlined that those tools mostly relied on the use of experimental or adapted microswitches capable of effectively and reliably monitoring the small responses that the participants could produce (41).

From a practical standpoint, two considerations are in order. First, these tools were not designed to be easily portable. Indeed, while the microswitches were readily wearable and portable, the computer to which they were connected was not necessarily easy to carry around. The lack of tools' portability is not deemed a real drawback in this context, given that the participants are confined in bed or in a wheelchair. The second consideration is that one may need to look at these tools as temporary solutions for a number of participants. For example, some participants with neurodegenerative diseases (e.g., amyotrophic lateral sclerosis) may lose the responses on which the tools initially relied and require the identification of new responses and new microswitches. Participants with acquired brain injury may improve their general level of functioning over time, and thus require more advanced support tools.

New research could be directed at (a) identifying additional microswitches that would allow the possibility of monitoring a larger variety of minimal responses with reliability and low intrusion, and (b) developing and assessing portable support tools. With regard to the latter objective, for example, one could conceive the use of smartphones for both monitoring specific responses (i.e., via the proximity and light sensors) and delivering stimulation contingent on the responses. The smartphones could be automated through available applications (e.g., MacroDroid) so as to control the specificity and duration of the stimulation in relation to the responses (46).

### Functional Body Movements

#### Studies

The four studies summarized in this section (16, 44, 46, 48) were aimed at individuals who could perform functional body movements and developed and assessed technology-aided support tools directed at promoting those movements. In particular, Shih and Chiu (48) set up a support tool to promote walking in place for two adolescents, who were affected by severe or mild intellectual disability and obesity, and tended to be largely passive. The technology used to detect the participants' step responses was a standard dance pad. The pad was connected to a computer device that could switch on and off specific videos considered to be preferred for the participants. In practice, the system turned on the videos when two sensor sections of the pad were activated in succession (i.e., as expected to occur during walking) and kept the videos on, as long as the sequential activation continued. Conversely, the system would not turn on, or would turn off the videos when the two sensor sections were simultaneously activated (i.e., as it occurs when an individual stands still). Data showed that the participants' levels of walking (i.e., step frequencies) increased drastically during the intervention when the support tool was in use.

Stasolla et al. (44) set up and assessed a support tool that was aimed at promoting assisted ambulation in two children, who were considered to function within the severe/profound intellectual disability range, had no speech abilities, and were performing only a few steps when provided with a walker device. The technology used to detect the participants' step responses consisted of a photocell, which was fixed onto the low lateral frame of the walker device and faced a reflecting panel. Any time the participant performed a step (moved the foot forward), the light beam produced by the photocell did not reach the reflecting panel and thus was not reflected back to the photocell. This lack of beam return triggered an electronic control device, which in turn activated 3 s of preferred stimulation (e.g., music and familiar voices). Data showed that the participants' step frequencies increased considerably during the intervention with the support tool and they were also accompanied by increases in indices of happiness.

Lancioni et al. (16) set up and assessed a support tool for promoting leg lifting movements in 11 participants with advanced Alzheimer's disease, who were sedentary and largely static particularly with regard to their lower limbs. The leg responses were monitored through one or two commercial tilt sensors/microswitches, which were linked to a computer device. Leg responses activated the microswitch(es), and in turn the computer, which delivered 10 s of preferred stimulation. The computer would provide a verbal encouragement to respond if the participant was passive for a preset period of time. Results showed that all participants had a clear increase in response frequency [thus, reaching a useful level of physical activity (49, 50)], which was accompanied by indices of positive participation/mood.

Lancioni et al. (46) set up and assessed a support tool aimed at fostering two functional responses (e.g., arm stretching to push a panel and leg-foot forward moving to push a box) for each of seven participants, who were characterized by severe or moderate/severe intellectual disability and extensive motor impairment confining them to a wheelchair. The technology used for monitoring the responses and providing stimulation contingent on their occurrences consisted of a smartphone device with Android operating system. The smartphone's functioning was regulated through the MacroDroid application, which ensured the recording of the responses and the delivery of 10 s of preferred stimulation for each response occurrence. With the use of the support tool, all participants showed large increases in response frequencies and indices of happiness. They also showed significant increases in heart rates, indicating that response performance represented a beneficial level of physical exercise (49, 51).

#### Comments on the Support Tools

The support tools reported for promoting functional body movements (a) relied on four types of response sensors (i.e., dance pad, photocell, tilt microswitch, and smartphone), (b) served participants with different levels of cognitive and motor functioning, and (c) focused on different responses. From a technical standpoint, one can underline the fact that the sensors on which the support tools relied were all commercially available and readily accessible in terms of costs. It may also be emphasized that while the dance pad, the photocell and the tilt device were connected to a computer system, which was to deliver stimulation, the smartphone had the dual function of sensor and stimulation device. Using a single instrument like a smartphone rather than two or more instruments (sensors and computers) would be seen as advantageous and could be easily viable in interventions such as those targeting footstep responses or leg movements.

From a practical standpoint, one can (a) reflect on the lack of portability of the support tools except the one relying on the smartphone, and at the same time (b) argue that portability might be a relatively marginal aspect given that the tools are typically employed in specific intervention settings rather than across settings. It might also be noted here that the advantage of using a single instrument like the smartphone should not be taken to suggest that its employment would be immediate (i.e., it would not require an appropriate preparation through suitable applications, such as the MacroDroid).

Future research may focus on extending the intervention to new functional movements, to new sensors, and eventually new support tools that would be easy to arrange in addition to being commercially available. For example, one might consider the possibility of targeting functional movements such as pulling oneself to a standing position and remaining in that position for brief periods of time (52) for people with congenital or acquired motor impairments with or without serious intellectual disability. Similarly interesting might be any initiative to foster those movements through support tools based on smartphones and thus commercially available and completely portable.

## Support Tools to Promote Functional Tasks

People with mild to severe intellectual disabilities with or without additional sensory or motor impairments, people with neurodegenerative diseases, in particular mild and moderate Alzheimer's disease, as well as people with acquired brain injury are often unable to carry out functional, multistep activities (21, 22, 35, 53). Indeed, they may fail to perform self-care sequences (e.g., morning routine), daily domestic tasks (e.g., preparing coffee, setting the table, or making a snack), and work tasks (e.g., assembling and packaging commercial products) (35, 54, 55). An increasingly popular approach to help these people concerns the use of technology-aided support tools providing step instructions and other types of assistance (56, 57). These tools can be defined as forms of cognitive prostheses bridging the gap between the people's skill level and the task demands (6, 58).

Six recent studies adopting a technology-based approach are summarized in this section (19, 21, 35, 59–61). The support tools used for helping the participants to perform the tasks varied on multiple aspects. Yet, the studies reported below are divided into two groups based on only one of those aspects, that is, on whether the support tools were non-portable (19, 60, 61), or portable (21, 35, 59) (see Table 2).

**Table 2:** Studies using support tools to promote functional tasks.

**Portability/Authors**	**Participants**	**Age**	**Support tools**
	**number**		
**NON-PORTABLE**			
Mihailidis et al. (19)	4	—	LCD touchscreen for verbal and visual instructions, cameras for monitoring participants' responses and animated job coach for feedback to support an assembly task
Lin et al. (60)	3	17	Two dance pads monitoring the participants' position and two computers presenting step instructions to support a domestic task
O'Neill et al. (61)	24	—	Computer with voice tracker and speech recognition to emulate staff prompting and questions to support morning routine
**PORTABLE**			
Cullen et al. (59)	3	20–24	iPad 4 with MyPicTalk application ensuring presentation of step instructions to support a cleaning task
Lancioni et al. (21)	8	64–79	Tablet with Android operating system and talking alarm set up to present time reminders and step instructions for multiple tasks
Lancioni et al. (35)	8	19–57	Smartphone with Android operating system and easy alarm youtube set up to present time reminders and step instructions for multiple tasks

### Tasks With Non-portable Support Tools

#### Studies

Mihailidis et al. (19) developed and tested a new technology-aided prompting/instruction tool to help individuals with intellectual and developmental disabilities carry out an assembly task. The core technology included a LCD touchscreen that provided the instructions verbally and visually (via pictures and videos), cameras for monitoring the participant's performance, an animated job coach providing encouragement and positive stimulation, and a complex software package. This technology system was able to determine the appropriate type of instruction to be presented to the participant based on his or her performance. The prompts/instructions could become more detailed (e.g., including extra visual elements), and plausibly helpful, based on the participant's difficulties. The timing of the instructions could also change based on the participant's progression. Whenever the technology system was unable to identify an instruction solution, a human job coach was alerted so that human supervision would be applied. The system was assessed in a pilot study with four adults with mild to moderate intellectual disability and a task including 18 steps. All adults showed an improvement in their task performance during the few intervention trials carried out.

Lin et al. (60) set up and assessed a technology-aided tool regulating video prompting to support activity skills in three adolescents with moderate intellectual disability. The technology involved two dance pads and two notebook computers. The notebooks were on two separate tables and the dance pads were placed before the tables. When the participant stood on the dance pad in front of the first table, the notebook on that table presented a video clip of the step response the participant was to perform (e.g., take the teacup) and accompanied the clip with the verbal instruction matching the video. When the participant moved to the second table and thus walked on the dance pad in front of it, the notebook on that table displayed the video of the step response the participant was to perform (e.g., put the teacup on the table, in the upper right corner) accompanying it with the matching verbal instruction. Then the participant was to continue to use the two tables with the two dance pads and notebooks until the task was completed. The task used for the three participants consisted of Chinese table setting and included 16 steps. With the support of the technology-aided tool, the participants managed to perform all 16 steps correctly with only sporadic exceptions.

O'Neill et al. (61) reported the set up and assessment of a micro-prompting device (“Guide”) to support the morning routine of people with acquired brain injury. The Guide system involved a computer with voice tracker, speech recognition software, activity protocols and activity protocol player. In essence, the computer involved audio-verbal interactive micro-prompting that was to emulate the verbal prompts and questions normally delivered by staff. Twenty-four adults with acquired brain injury were recruited for the study and randomly assigned to either the experimental (technology-aided) group or the control group. The morning routine sequence included seven main steps (e.g., getting up, showering, shaving, and dressing) and was supported via a plurality of step-related checks (questions) and instructions/prompts by the system. Data indicated that the experimental group required a significantly smaller number of direct staff interventions than the control group for carrying out and completing the morning routine accurately.

#### Comments on the Non-portable Support Tools

The support tools described above were developed with the objective of providing high levels of supervision and/or specific forms of interaction (closely simulating staff interaction) so as to ensure high levels of correct performance. The tool developed by Mihailidis et al. (19), in particular, was also capable of reorganizing the instruction sequence and the amount and type of instruction guidance so as to lead the participant to complete the task in a satisfactory manner. The tools used by Mihailidis et al. (19) and by O'Neill et al. (61) were fairly complex in terms of design and components. A slightly different consideration can be made for the self-prompting tool reported by Lin et al. (60). In fact, while it involved two laptop computers and two dance pads with relative interfaces, its working was fairly simple and thus required minimal software arrangement.

From a practical standpoint, one can make at least two considerations. First, the tools developed by Mihailidis et al. (19) and O'Neill et al. (61) appear quite expensive and relatively difficult to manage [i.e., compared to the one reported by Lin et al. (60)], with a potentially reduced affordability for and applicability within many contexts. Second, all support tools were tested on a single task, although the components/steps of the task reported by O'Neill et al. (61) were rather large and composite. A narrow testing provides an evidence base that does not allow one to determine the versatility of the tools for other tasks.

New research might be focused on setting up and assessing simpler versions of the tools to find out whether one can still ensure significant levels of success with those simplified (more accessible) versions. Another research point could be the assessment of those new tools' versions over a number of relevant tasks so as to establish their overall applicability within rehabilitation and occupation/work contexts.

### Tasks With Portable Support Tools

#### Studies

Cullen et al. (59) developed and assessed a technology-aided support tool that was to help three young adults manage a cleaning task and improve their performance of similar (generalization) tasks. The participants were diagnosed with autism or intellectual disability and visual impairment or traumatic brain injury, but their IQs were in the mild or above the mild intellectual disability range. The technology consisted of an iPad 4 with the MyPicTalk application allowing the use of video clips with voice-over for the single task steps. Prior to the start of the intervention on the target task (i.e., cleaning a table which included 12 steps), the participants were trained on how to use the iPad and related application so as to ensure that they would be ready for self-directed video prompting. All three participants showed a drastic performance improvement on the target task. They also showed variable levels of improvement on three generalization tasks.

Lancioni et al. (21) set up and assessed a support tool aimed at promoting the performance of daily tasks of eight participants with mild or moderate Alzheimer's disease. The tool reminded the participants of those tasks at the appropriate times and provided them with verbal instructions concerning the steps of those tasks. The technology included a tablet with Android operating system as well as a wireless Bluetooth earpiece, which allowed the participants to receive reminders and instructions without carrying the tablet. For each participant, 12 or 14 daily tasks, including means of 14 or 18 steps, were used. Six or seven tasks were scheduled per morning and/or per afternoon over periods of 2 or 3 h. When the time for a task was reached, the participant was reminded to start that task and thereafter he or she was presented with the instructions for it. With the use of the support tool, the participants managed to start virtually all the tasks scheduled independently, and reached high percentages of correct step performance.

Lancioni et al. (35) designed and assessed a support tool for promoting the performance of daily tasks of eight participants with mild/moderate or moderate intellectual disability and visual or hearing impairments. The technology consisted of a smartphone with Android operating system, which included standard functions and was fitted with the Easy Alarm YouTube application as well as with audio and video files for the single tasks. As soon as the time scheduled for a task was reached, the smartphone emitted a verbal reminder or a visual and vibratory reminder. The reminder was then followed by each of the step instructions (verbal or visual) arranged for the task. Ten to 12 tasks of 20–25 steps were available for each participant. Six tasks were scheduled per morning and/or per afternoon. With the use of the support tool, all participants managed to start the tasks at the appropriate times (following the reminders), and had high percentages of correct performance.

#### Comments on the Portable Support Tools

From a technical standpoint, the aforementioned support tools, which were used for a single task (59), or a plurality of tasks (21, 35), may be considered easily accessible. In fact, the tablets or smartphones and the applications required to automate their functioning are commercially available and readily acquirable. The availability of devices and applications should not be interpreted, however, as if all those support tools were ready for use by staff and caregivers. In fact, automating a tablet or smartphone to provide reminders and step instructions for a variety of tasks spread over specific periods of time requires a certain amount of preparatory work as well as a level of technical competence.

From a practical standpoint, four considerations may be in order. First, those support tools are easily affordable in terms of costs and thus suitable for daily contexts. Second, their usability for a variety of tasks makes them highly helpful in supporting the participants over large parts of the day, with minimal external supervision and maximum impact on the participants' functional engagement/interaction with their physical and social environment. Third, the tools are suitable for presenting verbal instructions as well as visual instructions. This versatility makes them appropriate also for people who have hearing impairment and/or verbal comprehension problems. Fourth, participants who use only verbal reminders and instructions do not have to carry the smartphone or tablet with them. Indeed, they can receive the reminders and instructions through wireless Bluetooth earpieces linked to those devices (21).

A primary objective of new research may be to gather additional evidence on the suitability and effectiveness of the aforementioned tools in (a) reminding participants with intellectual disabilities, Alzheimer's disease, and acquired brain damage about their daily tasks, and (b) supporting their performance of those tasks through verbal or visual instructions. Another research goal might be that of comparing the effectiveness of the tools' different instruction options (i.e., verbal, visual through static pictures, and visual through video prompts/clips) (62, 63).

## Support Tools to Promote Leisure, Communication, or Both

One common trait of many people with intellectual and other disabilities, neurodegenerative diseases, and acquired brain injury is the inability to engage in leisure activities independently (23, 37, 64, 65). This apparent inability may be largely due to difficulties in reaching and operating devices typically used to access leisure activities (e.g., television, computer, and music instruments). Another common trait concerns communication problems (29, 66–70). These problems may be characterized by the people's inability to (a) express their requests for caregivers and staff's attention or make other types of requests, and/or (b) reach relevant partners (e.g., preferred family or staff members) not immediately available in their context. The aforementioned problems may be related to lack of speech and alternative communication options and/or inability to use telephones or other devices to interact with distant partners (2, 37, 70–72). Studies have typically addressed one of the problems (i.e., either the leisure or the communication problem) at a time (1, 24, 29, 37, 73, 74). Recently, some studies have also been reported, which have addressed both problems within the same intervention program, thus allowing the participants to freely switch between the two types of engagement (32, 72).

The eight studies summarized in this section represent illustrative examples of interventions with support tools relevant for this area (see Table 3). Specifically, the first two studies addressed the leisure problem (75, 76); the following two studies addressed the communication problem (1, 77); and the final four studies targeted both problems within the same intervention context (32, 72, 78, 79).

**Table 3:** Studies using support tools to promote leisure, communication, or both.

**Target area/**	**Participants**	**Age**	**Support tools**
**Authors**	**number**		
**LEISURE**			
Stasolla and De Pace (76)	2	12, 14	Computer system presenting leisure options and a microswitch for choosing among those options and the alternatives they included
Lancioni et al. (75)	11	71–96	Computer system presenting leisure options and a microswtich for choosing among those options and the alternatives they included
**COMMUNICATION**			
Simacek et al. (77)	2	27, 7	One conventional speech-generating device requiring a touch response to be activated and an eye-tracking communication device working through eye-gaze responses
Davies et al. (1)	37	18–55	Hand-held speech-generating device whose screen images changed automatically across settings to facilitate adequate requests
**LEISURE AND COMMUNICATION**			
Borgestig et al. (78)	10	1–15	Eye-tracking communication devices allowing the use of eye gazes for communication and leisure/occupation
Lancioni et al. (32)	7	47–75	Computer system with a microswitch allowing choice among and supporting multiple leisure and communication options
Lancioni et al. (72)	8	35–58	Smartphone with Android operating system, which allowed the participants to access leisure events and telephone calls through the use of cards or mini objects fitted with frequency code tags
Lancioni et al. (79)	8	25–66	Tablet with Android operating system, which allowed the participants to access leisure events and video calls by simple hand responses

### Leisure

#### Studies

Stasolla and De Pace (76) reported the use of a support tool with two participants who had emerged from a minimally conscious state, and presented with extensive motor impairment, and lack of communication and interaction with the environment. The technology included a computer connected to a microswitch (i.e., a touch-sensitive device) through a specific interface. The computer would present visually and verbally different stimuli considered to be preferred for the participants (e.g., songs and mother's voice). The stimuli were presented in sequence and the participants could choose the one they wanted to access through microswitch activation. The selection of a stimulus led to the computer's presentation of several variations of that stimulus so that the participants could be more specific in their final choices and access what they most preferred at the time. During the intervention with the support tool, both participants managed to make choices thus accessing their preferred stimuli independently.

Lancioni et al. (75) set up and assessed a support tool, which technically resembled that used by Stasolla and De Pace (76), to enable 11 participants with mild or moderate Alzheimer's disease to engage in leisure activities independently. The participants did not have the ability to operate a computer or other device for leisure engagement. Yet, they discriminated verbal questions/instructions and visual images concerning preferred people and events, and were capable of activating a pressure microswitch for operating their choices. The technology (a) included a laptop computer with screen and sound amplifier, which was linked to a pressure microswitch, and (b) allowed the participants to choose among music, comedy, films, and television shows. The participant could choose any option by activating the microswitch when that option was highlighted. After choosing an option, the participant was presented with a variety of stimuli connected to it, so he or she could select the one to access. During the intervention (i.e., when the support tool was available), all participants displayed successful choice performance, thus accessing a variety of stimuli and remaining positively engaged throughout most of the time allotted.

#### Comments on the Support Tools

Individuals whose condition (e.g., intellectual and motor disabilities or Alzheimer's disease) precludes them from reaching/accessing preferred stimuli and engaging with them freely need the help of a support tool to bypass those limitations. The tools described above were aimed at supporting individuals with traumatic brain damage and individuals with Alzheimer's disease. Similar tools have also been used with people with intellectual and multiple disabilities, with amyotrophic lateral sclerosis or various forms of brain injury (37, 80). From a technical standpoint, the aforementioned tools can be considered relatively simple as they included (a) a computer presenting the stimuli available for choice and delivering the stimuli that the individuals eventually chose, and (b) a microswitch connected to the computer that allowed the participants to carry out their choice responses. Although rather simple, those tools are not readily accessible/available and need to be prepared for the single participants so as to respond to their preferences. It is also noteworthy that experimental microswitches may be needed for participants whose motor repertoire is very poor (80).

From a practical standpoint, one can consider the tools fairly friendly for the participants, manageable for staff and caregivers, and reasonable in terms of costs. A possible question as to their accessibility for daily contexts may arise whenever an experimental microswitch is required. The fact that those tools are not always easy to carry around may not be a serious drawback given that most of the individuals using those tools are confined to specific contexts, and thus do not need to carry the tools across settings (2, 24).

New research could focus on arranging and testing new technology packages so as to have a range of different solutions to address the needs of individuals with different characteristics. It is also important to recognize that support tools might be more valuable if they do not only allow leisure engagement, but also provide the conditions for communication (see below) (72, 79). Tools supporting different forms of engagement would promote performance variability and thus ensure longer periods of profitable occupation.

### Communication

#### Studies

The main body of intervention studies concerning communication have addressed the participants' inability to make (verbal) requests and assessed the impact of tools such as speech-generating devices (e.g., iPods and iPads) (26, 28, 67, 68). The two studies summarized below (1, 77) seem to add to the main body of the literature available in terms of technology used and/or participants involved. In particular, Simacek et al. (77) arranged two types of communication support tools to foster request making in two participants with Rett syndrome. The older participant had a diagnosis of atypical Rett syndrome and was able to ambulate, although with assistance, and could use her hands to press, grasp, hold, and release objects. The younger participant had a diagnosis of typical Rett syndrome, spent most of her time in a sitting position, and did not manipulate objects. She was reported, however, to gaze at objects she presumably wanted. The technology consisted of (a) a speech-generating device that could be activated by touching the image of the object requested (older participant) or (b) an eye-tracking device that could be activated by looking at the object requested for a prefixed amount of time (younger participant). Both participants were taught to request three preferred items through various intervention steps that also involved increasing the size of the images of the preferred items. Data showed that each participant learned to use the relative communication support tool to acquire requesting for the three preferred items. The older participant was relatively fast in her acquisition. The younger participant needed more time. Yet, she seemed to represent the first case with Rett syndrome to acquire multiple requests through an eye-gaze response.

Davies et al. (1) set up and assessed a new technology system (i.e., GeoTalk) as support tool for facilitating request making across different settings with a group of 37 people. The people's average IQ score was in the low region of the mild range. The technology involved a hand-held communication device that integrated a global positioning system (GPS) and other sensors, which allowed the vocabulary/communication symbols appearing on the device's screen to change automatically based on the geographic zone. When the participant was in a zone such as the school, the symbols were those normally used for communication requests made in that zone. If the participant moved to a grocery store, the symbols available on the device changed accordingly. To determine the usability and effectiveness of the GeoTalk, the participants' performance with such tool was compared with their performance with two other communication technology solutions. One involved a speech-generating device in which, the participant was to change the communication-symbol layer independently when he or she entered a new context. The other involved a device based on a palmtop computer in which the symbol sets were to be changed through a screen operation. Data showed that the GeoTalk compared favorably with the other devices. The participants made fewer errors, required fewer prompts, and needed shorter time to make the requests.

#### Comments on the Support Tools

The support tools described above, although generally defined as speech-generating devices, differ from those previously available in the area in that they include components that can make their use more effective (1) and/or feasible also for people with no reliable hand movements (77). From a technical standpoint, the tools reported [except for the one used by Simacek et al. (77) for the older Rett participant] can be considered relatively complex. Essentially, the tool reported by Davies et al. (1) is an experimental arrangement of available technology components, which was specifically designed to assist people across settings. The eye-gaze tool reported by Simacek et al. (77) for the younger Rett participant is based on commercial eye-tracking technology, which (a) supports multiple activities besides communication, and (b) is typically used with individuals with amyotrophic lateral sclerosis or other conditions of pervasive motor impairment.

From a practical standpoint, two considerations are in order. First, those tools are not designed to be easily portable. Indeed, the Davies et al.'s tool involves a number of sensors that are tied to specific settings and work properly within those settings. The tool reported by Simacek et al. for the younger participant involves a relatively sophisticated computer-aided system that is generally fixed to a table stand or the participant's wheelchair and cannot always be freely moved across settings or used across individuals. Second, the cost of those tools is expected to be considerable and their set up requires a certain amount of competence.

New research work could advance the development of the Davies et al.'s tool with new/cheaper commercial components (e.g., with smartphones functioning as speech-generating devices) so as to make it more affordable and easier to set up and use as a real resource for individuals attending school, work and other community settings. A simpler and cheaper eye-gaze device may also need to be developed (to replace the expensive commercial versions; e.g., Tobii C12) specifically for individuals who only use it for limited purposes.

### Leisure and Communication

#### Studies

Borgestig et al. (78) set up and assessed eye-tracking support tools for 10 participants who presented with extensive motor impairments. Five of them were also reported to have an unspecified level of cognitive impairment. All participants were said to be able to communicate (show interest) with facial expressions and eye gazing. Some participants could also express yes/no eye movements. The technology consisted of Tobii C12 or Tobii P10 eye-tracking devices mounted on a floor stand, table stand, or the wheelchair. Following a protracted intervention phase, during which parents, teachers and technology experts were involved in promoting the use of the technology, there was a follow-up assessment during which expert supervision was no longer available. Follow-up data showed that all 10 children managed to use the eye-tracking devices. Most children learned to talk with others via the devices. However, communication was the main purpose in using the devices only for two children. Other children used the devices predominantly or exclusively for playing games, watching photos, or listening to music.

Lancioni et al. (32) set up and assessed customized support tools to meet the leisure and communication needs of seven participants with acquired neurological damage and multiple disabilities. Participants presented with lack of expressive communication and pervasive motor disabilities that prevented them from having any direct, independent contact with environmental stimuli. The technology involved a computer, which (a) showed the leisure and communication options available for the single participants (e.g., songs, television, direct requests, text messages, or writing) and (b) supported any of those options, thus allowing the participants to access and engage in any of them. For example, if the participant chose text messages, the computer presented (a) the names of various persons to whom messages could be sent, and (once a person was selected), (b) the messages available for that person. The participants could make their choices and manage any leisure or communication event through the use of a microswitch suiting their motor condition. All participants managed to use the support tools successfully, and consequently engaged in leisure and communication consistently throughout the study.

Lancioni et al. (72) set up and assessed a support tool that was aimed at promoting leisure activities and telephone calls in eight participants who presented with mild to moderate intellectual disability and sensory or sensory-motor impairments. The technology consisted of a smartphone with Android operating system, which was (a) supplied with audio or audiovisual files concerning the leisure activities and telephone partners for communication and (b) automated through the MacroDroid application. The participants made their requests for leisure activities or telephone calls by placing cards or mini objects fitted with frequency code tags on the back of the smartphone. Recognition of the cards and mini objects' tags led the MacroDroid to activate the related activities or telephone calls thus allowing the participants to access them. With the help of the support tool, all participants learned to make requests and access leisure events and telephone calls successfully and maintained their positive performance over time.

Lancioni et al. (79) set up and assessed an additional support tool to ensure access to leisure activities and video calls to eight participants who presented with moderate intellectual disability and had very poor speech skills or had no speech and no receptive verbal skills due to hearing loss. Video calls seemed the only way or the preferred way for the participants to interact/communicate with distant partners. The technology involved a tablet with Android operating system, which was fitted with a SIM card and two specific applications, that is, WhatsApp Messenger for making video calls and MacroDroid for automating the functioning of the tablet. The tablet typically presented pictures representing leisure activities and pictures concerning preferred partners. The participant could select any of them by touching/approaching the tablet's proximity sensor when the activity or partner was lit. Selection of an activity led the tablet to present several variations of it among which the participant could choose. Selection of a partner led the tablet to start a video call with that partner. Data showed that all participants learned to use the support tool and were successful in accessing leisure activities and making video calls independently.

#### Comments on the Support Tools

Four different support tools were described in this section, that is, commercial eye-tracking devices, a combination of computer system and microswitch, a specially arranged smartphone, and a specially arranged tablet. From a technical standpoint, the tools differ substantially, with the first two being much more complex than the other two. Indeed, the eye-tracking devices are highly sophisticated and powerful instruments that require a careful tuning with the participants under expert supervision, and thus are not readily/immediately accessible. The microswitch used in combination with the computer system is frequently an experimental device or an adaptation of a commercial device (i.e., to suit participants affected by pervasive motor impairment), and this requires extra preparation time and costs. The last two tools are based on common commercial technology and thus are more easily accessible and comparatively simple. Even so, adapting them for the participants' use requires, as suggested in previous sections of this paper, a certain amount of preparation work and expertise involving the management of specific applications, such as the WhatsApp Messenger and the MacroDroid.

From a practical standpoint, a few considerations are in order. Eye-tracking devices may not be easily affordable in daily contexts given their complexity and costs. Their employment with individuals with pervasive motor disabilities might often be successfully replaced by the use of support tools involving the combination of computer and microswitch. These latter tools, albeit not immediately accessible (as observed above), (a) can be adapted to individuals with minimal response repertoire, and (b) are much less expensive than the eye-tracking devices. The other two support tools, based on smartphone and tablet technology, have the advantage of being fairly inexpensive/affordable and easily portable. Yet, they are applicable only when the participants have control of the basic motor responses necessary to manipulate cards or mini objects and to activate the tablet's proximity sensor, respectively.

New research may be focused on the development of alternative support tools that can promote leisure activities, audio and video calls, and message exchanges, and are easily affordable and portable. One might also explore the possibility of using smartphones as microswitches for participants with no use of their hands (81). For example, a smartphone could (a) monitor, through its light or proximity sensors, small movements of the participant's head, and (in relation to those movements) (b) operate leisure or communication choices on a second smartphone or tablet.

## Conclusions

The paper has analyzed a number of support tools, which were used to help people with significant disabilities bypass the limits imposed by their condition and reach important objectives. The generally positive results reported by the studies summarized above are encouraging as to the beneficial role of those tools. They were successfully used (a) as extension of the individuals' body, so the individuals could engage with the environment effectively irrespective of their response limits, and (b) as extension of the individuals' cognitive dimension, so the individuals could act in a more accomplished manner and improve their occupational achievement, social contacts and communication. In essence, technology-based support tools were reported to be instrumental in fostering goal-directed interactions of the individual with disability with his or her physical and social environment, and possibly in promoting the individual's cognition and development (8–10, 82, 83). Notwithstanding the above, some caution might still be needed in drawing general conclusions, due to the fact that (a) the studies reported to illustrate the tools included few participants, (b) the relatively limited data available do not allow sophisticated statistical analyses and do not provide specific evidence of the tools' use in daily contexts (i.e., under the supervision of regular staff), and (c) the rapidly changing field of technology might shortly present new scenarios with new, alternative tools.

The appropriateness and friendliness of any tool are high when the tool's operation fits the individual's physical conditions and/or cognitive skills and when the goal set to be reached is feasible for the individual [i.e., in line with his or her embodied experience (84)]. In light of this statement, one could also maintain that support tools are to be adapted in terms of their operation requirements and/or their content (the goal set to be reached) to the single individuals exposed to the tools (85, 86). Adaptations/customizations would ensure that all individuals are able to manage the use of the tools provided and reach the results expected easily and rapidly (i.e., without failures and frustration and without physical strain) (32).

One additional element that would have a decisive impact on the final results obtained with any tool is the individual's motivation to use such tool (i.e., the individual's motivation to reach the input/consequences available for the tool-mediated performance). The individual may be strongly motivated to produce high response rates (e.g., high levels of small responses or functional body movements) if the stimulation following the performance of those responses is significant (highly preferred) for him or her (12, 21, 42, 87). Similarly, the individual may be strongly motivated to have high levels of task accuracy and high levels of leisure and communication engagement if his or her performance encounters success and satisfaction. Satisfaction could be here interpreted as (a) the availability of positive feedback (higher level of social enclosure) for correct performance, (b) the possibility to access leisure events meeting the individual's interests, and (c) communication opportunities involving the individual's preferred partners and preferred topics (2, 87).

Acceptance and regular use of support tools within daily contexts cannot be considered an easy-to-reach objective for a variety of reasons, some of which were discussed above. Indeed, one would assume that accessibility, affordability and friendliness of the tools represent main variables favoring their application outside of the research environment. The presence of all these variables alone, albeit essential, might not yet guarantee a positive decision of daily contexts in relation to the use of support tools. There are situational/practical issues, in fact, that may interfere with a positive decision (88–91). A major issue is the knowledge (competence) gap that exists between the experimental world in which tools are developed and assessed and the daily reality. The gap cannot be bridged by simply relying on technology experts, who have no specific competence in the field of education and rehabilitation, and thus cannot ensure the identification of a suitable tool for each single individual (32, 72). The gap may be reduced, however, when technology experts are called to work in close collaboration with education/rehabilitation personnel who can identify the skills and limitations of the individuals to serve and the objectives to target with them.

In conclusion, the support tools presented and discussed above seem to have great potential for improving the situation of individuals with serious disabilities by providing those individuals with new opportunities for (a) engaging in goal-directed interaction with the environment and thus (b) enhancing cognition and development. The possibility of having those tools largely available in daily contexts may greatly depend on (a) the accessibility, affordability, and friendliness of the tools, and (b) the availability of areas of competence and responsibility in the daily contexts that would ensure a successful set up and application of the tools, and possibly a positive intervention outcome.

## Author Contributions

GL was responsible for conceiving and writing the paper. MOB, NS, MO, JS, and GA were involved in defining the scope of the paper and the material to include, and contributed in the writing and editing of the manuscript.

### Conflict of Interest Statement

The authors declare that the research was conducted in the absence of any commercial or financial relationships that could be construed as a potential conflict of interest.
